# Differences in Microbiota Membership along the Gastrointestinal Tract of Piglets and Their Differential Alterations Following an Early-Life Antibiotic Intervention

**DOI:** 10.3389/fmicb.2017.00797

**Published:** 2017-05-09

**Authors:** Chunlong Mu, Yuxiang Yang, Yong Su, Erwin G. Zoetendal, Weiyun Zhu

**Affiliations:** ^1^Jiangsu Key Laboratory of Gastrointestinal Nutrition and Animal Health, Laboratory of Gastrointestinal Microbiology, College of Animal Science and Technology, Nanjing Agricultural UniversityNanjing, China; ^2^Laboratory of Microbiology, Wageningen UniversityWageningen, Netherlands

**Keywords:** early-life antibiotics, gut microbiota, compartmentalization, stomach, small intestine, microbial function

## Abstract

Early-life antibiotic interventions can change the predisposition to disease by disturbing the gut microbiota. However, the impact of antibiotics on gut microbiota in the gastrointestinal tract is not completely understood, although antibiotic-induced alterations in the distal gut have been reported. Here, employing a piglet model, the microbial composition was analyzed by high-throughput 16S rRNA gene sequencing and PICRUSt predictions of metagenome function. The present study showed clear spatial variation of microbial communities in the stomach and intestine, and found that the administration of antibiotics (a mixture of olaquindox, oxytetracycline calcium, kitasamycin) in early life caused markedly differential alterations in the compartmentalized microbiota, with major alterations in their spatial variation in the lumen of the stomach and small intestine. In piglets fed an antibiotic-free diet, most of the variation in microbial communities was concentrated in gut segments and niches (lumen/mucosa). The microbial diversity was higher in the lumen of stomach and duodenum than that in ileum. The early-life antibiotic intervention decreased the abundance of some *Lactobacillus* species and increased the abundance of potentially pathogenic *Streptococcus suis* in the lumen of the stomach and small intestine. Interestingly, the intervention increased the abundance of *Treponema* only in the colonic lumen and that of *Faecalibacterium* only in the ileal mucosa. Furthermore, the antibiotic intervention exerted location-specific effects on the functional potential involved in the phosphotransferase system (decreased sucrose phosphotransferase in the stomach) and antibiotic-resistance genes (increased in the colon). These results point to an early-life antibiotic-induced dramatic and location-specific shift in the gut microbiota, with profound impact in the foregut and less impact in the hindgut. Collectively, these findings provide new insights into the membership of the microbiota along the gastrointestinal tract of piglets and highlight the importance of considering the foregut microbiota in health management of piglets at early life.

## Introduction

Gut microbiota are important to the metabolism and health of their hosts. The composition and distribution of the microbiota of the intestine of pigs have been extensively investigated (Kim and Isaacson, 2015). Relative to the large intestine, less information is available on the microbiota in the stomach and small intestine. Small intestinal bacteria actively participate in the digestion and metabolism of carbohydrates (El Kaoutari et al., 2013) and amino acids (Dai et al., 2010, 2012).

Recent studies have shown that the distribution of microbiota in the gastrointestinal tract (GIT) of the pig varied between small intestine and large intestine and between lumen and mucosa (Looft et al., 2014a). In growing pigs, *Proteobacteria* and *Firmicutes* dominated in the digesta of ileum while *Bacteroidetes* and *Firmicutes* dominated in the digesta of colon (Looft et al., 2014a; Zhao et al., 2015). The microbial composition differs in lumen and mucosa of small intestine, characterized as the higher *Anaerobacter* and lower *Prevotella* in the lumen than mucosa in ileum (Looft et al., 2014a). In addition, our previous studies found that the cultured luminal and mucosal microbes from porcine gut had different role in amino acid metabolism (Yang Y.X. et al., 2014). These suggest that microbiota from different intestinal compartments and niches have different functional roles. It is widely regarded that compared to the microbial numbers in the colon (10^11^–10^12^ CFU/mL), stomach and small intestine have relatively low numbers of bacteria, with 10^1^–10^3^ CFU/mL in the lumen of stomach and duodenum, and 10^4^–10^7^ CFU/mL in the lumen of jejunum and ileum (O’Hara and Shanahan, 2006). Thus, the microbiota in the stomach, duodenum, and jejunum has received little attention. In our previous study investigating the change of bacterial community of the piglets around weaning using denatured gradient gel electrophoresis method, the bacterial community in the stomach was markedly different with that in the jejunum and ileum before weaning, but became highly similar after weaning, characterized with an increase in *Streptococcus suis* both in the stomach and small intestine (Su et al., 2008). Thus, to gain further insight into the distribution of gut microbiota, it is crucial to understand the spatial variation in microbiota across the stomach to intestine.

Research showed that an early-life antibiotic intervention influenced host health by altering the gut microbiota (Zeissig and Blumberg, 2014). Early-life intervention of tulathromycin on day 4 after birth increased *Faecalibacterium prausnitzii* and *Eubacterium* in the jejunal content at day 8 of age in piglets (Schokker et al., 2014), suggesting an important impact of early-life antibiotic intervention on gut microbiota. However, the effects of antibiotics on the gut microbiota may vary in different gut locations, but studies of this type are limited. In growing pigs, in comparing gut microbiota among ileum, cecum, and colon, Looft et al. (2014a) found an in-feed antibiotic mixture of chlortetracycline, sulfamethazine, and penicillin, showed different effects among the gut segments. After the antibiotics, *Escherichia* increased in the lumen of ileum, cecum, and colon, while *Treponema* decreased in the lumen of cecum, colon, and feces (Looft et al., 2012, 2014a). In piglets, limited research showed that in-feed chlortetracycline reduced the counts of *Lactobacillus johnsonii* in the lumen and mucosa of ileum (Rettedal et al., 2009), but had no effect on fecal microbiota composition (Poole et al., 2013). These results suggest that the effects of antibiotics on gut microbiota could vary in small versus large intestine. The stomach and duodenum are the first organs in the GIT that contacts orally administrated antibiotics. Therefore, it is important to understand the simultaneous response of the stomach and intestine to orally administrated antibiotics.

In the present study, the goal was to characterize the microbial composition along the GIT of piglets, especially for stomach and duodenum, and the effects of early-life antibiotics on the microbiota along the GIT (from stomach to colon). The hypothesis was that early-life antibiotics may differentially affect the microbial community structure in the foregut. We focused on the early-life antibiotic intervention as in-feed antibiotics are regularly used for disease prevention and growth promotion during the early-life period (from day 7 to 42 of age), when piglets are sensitive to external pathogens. The results showed that although not as diverse as in the colon, the stomach and duodenum of piglets also had a highly diverse microbiota. This finding provides new insight into the membership of the microbiota along the GIT, with its function yet to be explored. Importantly, early-life in-feed antibiotics predominantly altered the microbial composition in the foregut rather than hindgut. These findings also implicate the importance to consider the foregut microbiota in health management of piglets.

## Materials and Methods

### Animal Husbandry

This study was carried out in accordance with the recommendations of the Guidelines for the Care and Use of Animals of Nanjing Agricultural University. The protocol was approved by the Ethical Committee of Nanjing Agricultural University, Nanjing (authorization number SYXK (Su) 2011–0036).

A total 16 litters of newborn piglets (Duroc × [Landrace × Large white]; 187 in total) were allotted to control group and in-feed antibiotic group, with 8 litters per group. At day 7, while staying with their respective sows and sucking sow milk, the piglets started to receive creep feed containing either no antibiotics (control group) or antibiotics (antibiotic group). At day 23, all the piglets were weaned and transported to a nursery house in a controlled environment with 1.8 × 2.5 m pens (16 pens) fitted with a hard plastic fully slotted floor, and fed the same diet as creep feed with or without antibiotics until day 42 of age. For the antibiotic group, a mixture of antibiotics (50 mg/kg olaquindox, 50 mg/kg oxytetracycline calcium, 50 mg/kg kitasamycin; antibiotic treatment group) was added to the creep feed. Oxytetracycline calcium, an antibiotic that directly targets both Gram-positive and Gram-negative bacteria, and kitasamycin, a macrolide antibiotic that directly targets Gram-positive bacteria, and olaquindox, an antibiotic that improves feed efficiency, were chosen because a mixture of them are regularly used in piglets (Zhang et al., 2016). This combination of antibiotics is commonly used in China in feed for sucking and weaning piglets. The rationale behind the concentrations used was according to the dose limitation in Regulations of Feeding Drug Additives (announcement No. 168) approved by Ministry of Agriculture, China.

The creep feeding diet was based on corn and soybean-meal and designed to meet the requirement of NRC (2012), as shown in Supplementary Table S1. The piglets were fed twice daily (0800 and 1700 h, equal portions at each meal) and had free access to water via a low-pressure nipple drinker. Feed refusals were monitored and average daily feed intake was calculated.

### Slaughtering and Sampling

At day 42, after fasting overnight, one piglet from each litter was randomly selected for sampling. The GIT of each piglets was removed immediately after slaughter and segments (stomach, duodenum, jejunum, ileum, cecum, and colon) were identified and ligated before separation. Digesta of the stomach, duodenum, jejunum, ileum, and colon were collected and stored at -70°C until analysis. The mucosa was rinsed with sterile ice-cold phosphate buffered saline several times to remove the remains of free-floating bacteria. Mucosal samples from duodenum, jejunum, and ileum were collected by scraping off the mucosa using a sterile glass microscope slide. Mucosal scrapings were kept on ice until being stored at -70°C. A total of 128 samples from 8 gut locations of 16 piglets, were collected.

### DNA Extraction, 16S rRNA Gene Amplification, and High-Throughput Sequencing

Total genomic DNA in digesta (stomach, duodenum, jejunum, ileum, and colon) and mucosal scrapings (duodenum, jejunum, and ileum) were extracted from 0.3 g of sample using a bead-beating and phenol-chloroform extraction as previously described (Yang et al., 2016). The V3–V4 region of the 16S rRNA gene was amplified with universal bacterial primers (forward 5′-barcode-TAC GGR AGG CAG CAG-3′ and reverse 5′-AGG GTA TCT AAT CCT-3′) (Singh et al., 2015), where barcode is an eight-base sequence unique to each sample. The barcodes were set according to previous report (Fadrosh et al., 2014). PCR was carried out in triplicate using the 20 μL reactions containing 10 ng of template DNA, 2 μL of 10-fold reaction buffer, 1 μL DNA, 0.4 μL each primer, 1.25 U TaKaRa Ex Taq (Takara Biotechnology, Dalian, China), 1.6 μL dNTPs, and 14.35 μL ddH_2_O. Amplicons were extracted from 2% agarose gels and purified using the AxyPrep DNA Gel Extraction Kit (Axygen Biosciences, Union City, CA, USA) according to the manufacturer’s instructions and quantified using QuantiFluor -ST (Promega, USA). Purified amplicons were pooled in equimolar amounts and paired-end sequenced (2 × 250) on an Illumina MiSeq platform according to the standard protocols (Caporaso et al., 2012).

Raw sequence data were trimmed, filtered, aligned, and classified using Mothur software package (Ver 1.32.0) (Schloss et al., 2009). To improve the joining potential of the paired-end raw sequence reads, Trimmomatic was used to trim the low quality base at the overlapping end of reads and join reads by iterating over a 50 bp range of quality scores (1–20) as the threshold for removal. One sample from ileal lumen of the antibiotics-treated piglets was not analyzed due to unsuccessful sequencing.

### Bioinformatics Analysis of Sequencing Data

After trimming the primer, barcode, and chimeras, the unique sequences were identified and aligned against high quality 16S rRNA sequences from the Greengenes reference alignment (DeSantis et al., 2006). After screening, filtering, and pre-clustering processes, gaps in each sequence were removed in all samples to reduce noise. Using the average neighbor algorithm with a cutoff of 97% similarity, these sequences were clustered into operational taxonomic units (OTUs). Representative sequences from each OTU were taxonomically classified with a confidence level of 90% (Cole et al., 2009). Sequences that only occur once in all 127 samples were excluded as they could be spurious and may bias diversity estimations. For estimation of species diversity, spurious singletons were discarded and the remaining OTUs were used for calculations. Species richness estimator (Chao 1 and ACE), diversity indices (Shannon and Simpson), Shannon evenness (a Shannon index-based evenness), and Good’s coverage were calculated (Schloss et al., 2009). Unweighted Unifrac principal coordinate analysis (PCoA) (Lozupone et al., 2011) based on OTUs was performed to provide an overview of the gut microbiota composition in the different segments and the microbial response to antibiotics. Analysis of molecular variance (AMOVA) (Schloss et al., 2009) was performed to compare the difference between lumen and mucosa, antibiotic and control and adjacent gut segments.

Here we defined the segments to represent longitudinal locations (from stomach to colon) and niches to represent radial locations (from mucosa to lumen). To determine the impact of gut segments, niches (lumen/mucosa) and antibiotics on the variation observed, Redundancy analysis (RDA) was performed at OTU level using Canoco 5.0 software (Šmilauer and Lepš, 2014).

### Prediction of Microbial Metagenomes

The metagenomic prediction of the functional capacity based on 16S rRNA gene composition was done with PICRUSt (Langille et al., 2013). OTUs were normalized by copy number, and the gene categories were predicted at level 2 and level 3 KEGG orthology groups (KOs) using the Kyoto Encyclopedia of Genes and Genomes (Kanehisa et al., 2012). The metagenomic prediction can produce the KEGG IDs and Enzyme Commission IDs. KEGG IDs were used to search against the Comprehensive Antibiotic Resistance Database^1^ (McArthur et al., 2013), and the Enzyme Commission IDs were used to search against the Carbohydrate-Active Enzyme database^2^ (Lombard et al., 2014). The KOs were further mapped to KEGG modules using iPath 2.0 (Yamada et al., 2011).

### Statistical Analysis

Graphing and statistical analysis were performed using Graphpad Prism (La Jolla, CA, USA) and IBM SPSS Statistics software (version 20.0, IBM, Armonk, NY, USA). For comparisons between 2 groups, unpaired Student’s *t*-test for parametric data and Mann–Whitney *U*-test for non-parametric data were used. For comparisons between > 2 groups, one-way ANOVA with Bonferroni *post hoc* test for parametric data and Kruskal–Wallis ANOVA performed on ranks for non-parametric data were used. Significance was declared at *P* < 0.05. In case of multiple comparisons, *P*-values were adjusted with a false discovery rate (FDR) analysis (Benjamini and Hochberg, 1995), limiting the overall false discovery rate to 5% (*q* < 0.05).

### Accession Number

The sequence information in this paper has been deposited in the GenBank Sequence Read Archive database under accession number SRP102481.

## Results

The average daily feed intake per piglet was not statistically different (*P* > 0.05) between the control group (mean ± SEM: 515.6 ± 13.9 g) and antibiotic group (mean ± SEM: 516.2 ± 8.5 g; data not shown). Since the feed intake was similar among individual piglets in the antibiotic group, we assumed that the antibiotic consumption was also similar among the individual piglets.

### Summary of High-Throughput Sequencing Data

All the reads from high-throughput sequencing of 16S rRNA genes of all the samples at eight gut locations were processed together. In total, 3,888,709 sequences passed the quality control. On average, 30,381 sequences per sample were obtained, with an average length of 440 bp. Using the criterion of 97% sequence similarity at the species level, 2,019 OTUs were identified, all of which belonged to the bacteria domain according to Greengenes classification. Finally, on average, 267 ± 102 OTUs (Good’s coverage) per sample were identified.

### Impact of Gut Location, Lumen/Mucosa, and Antibiotics on the Bacterial Composition

**FIGURE 1:**
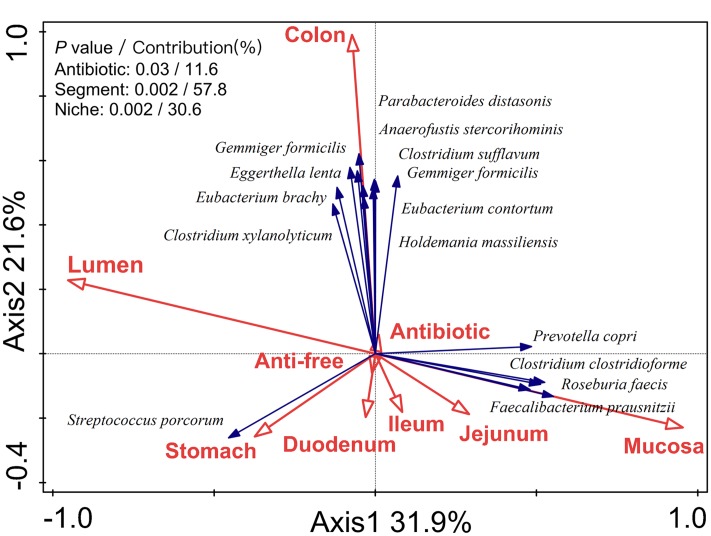
**Biplot of the RDA (redundancy analysis) based on the relative abundance of OTUs (97% similarity level).** RDA biplot aimed to present the impact of environmental variables on the microbiota variation and the relationship between species and environmental variables. Segments represented longitudinal locations (from stomach to colon) and niches represented radial locations (from mucosa to lumen).

To determine the impact of the gut segment (longitudinal locations from the stomach to the colon), niche (radial locations from lumen to mucosa), and antibiotics on the observed variation, RDA analysis was performed. The RDA analysis of the microbiota structure at the OTU level indicated that the antibiotic intervention (*P* = 0.03), gut location (*P* = 0.002), and lumen/mucosa (*P* = 0.002) all had a significant impact on the microbial composition in the piglets (**Figure 1**). Among these, the gut segment was mainly responsible for shaping the bacterial profile of the piglets (contribution = 57.8%), followed by the niche (lumen/mucosa, contribution = 30.6%) and antibiotic intervention (contribution = 11.6%).

**FIGURE 2:**
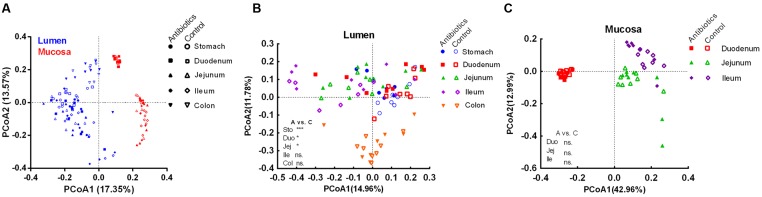
**Principle coordinate analysis of all samples including control and antibiotic group (A)**, luminal samples **(B)**, and mucosal samples **(C)** in the control and antibiotic group by unweighted Unifrac distance. The AMOVA significance was also indicated: ^*^P < 0.05; ^*^^*^^*^P < 0.001; ns, non-significant. Abbreviations: A, antibiotics; C, control; Col, colon; Duo, duodenum; Ile, ileum; Jej, jejunum; Sto, stomach.

To further reveal the impact of the antibiotic intervention on the microbiota composition, PCoA was performed based on unweighted (presence/absence information) Unifrac distances. Different samples clustered primarily by the niche (mucosa/lumen) (**Figure 2A**). In the control group, there was a significant difference in the microbiota composition of the lumen and mucosa and the microbiota composition of different mucosa sites (analysis of molecular variance [AMOVA], *P* < 0.05, **Table 1** and Supplementary Figure S1). Furthermore, the microbiota diversity of the stomach and duodenum was higher (*P* < 0.05) than that of the ileum, as measured by the Shannon index (Supplementary Figure S2A). Although the antibiotic intervention did not change the evenness of the gut microbiota (Supplementary Figure S2B), it significantly shifted the microbial structure of the lumen of the stomach, duodenum, and jejunum (AMOVA, *P* < 0.05; **Table 1** and **Figure 2B**). The antibiotic intervention did not alter the microbial structure of the mucosa of the duodenum, jejunum, or ileum (AMOVA, *P* > 0.05; **Figure 2C**). These findings confirmed the varied microbiota composition of segments versus niches. Furthermore, these results indicated that at the end of the experiment, early-life antibiotics had marked effects on the overall structure of gut microbiota in foregut lumen of piglets.

**Table 1:** Analysis of molecular variance analysis of microbiota content of different groups.

Item	Sum of square		Φ_ST_	*P*-value
	Among groups	Within groups		
**Control group**				
Lumen vs. Mucosa				
Duodenum	0.778	1.155	9.425	<0.001
Jejunum	0.986	1.388	9.941	<0.001
Ileum	0.821	1.291	8.260	0.001
Proximal vs. Distal				
Lumen				
Stomach-Duodenum	0.104	1.985	0.730	0.896
Duodenum-Jejunum	0.363	2.078	2.445	0.003
Jejunum-Ileum	0.208	2.086	1.296	0.186
Ileum-Colon	0.684	1.752	5.075	<0.001
Mucosa				
Duodenum-Jejunum	0.328	0.594	7.732	<0.001
Jejunum-Ileum	0.686	0.466	20.634	<0.001
**Antibiotic vs. Control**				
Lumen				
Stomach	0.260	1.859	1.957	<0.001
Duodenum	0.271	2.498	1.522	0.050
Jejunum	0.267	2.369	1.580	0.048
Ileum	0.205	2.880	0.925	0.488
Colon	0.174	1.742	1.405	0.092
Mucosa				
Duodenum	0.017	0.271	0.857	0.573
Jejunum	0.107	0.869	0.976	0.102
Ileum	0.076	0.565	1.895	0.057

### Bacterial Taxa Affected by the Early-Life Antibiotic Intervention

**FIGURE 3:**
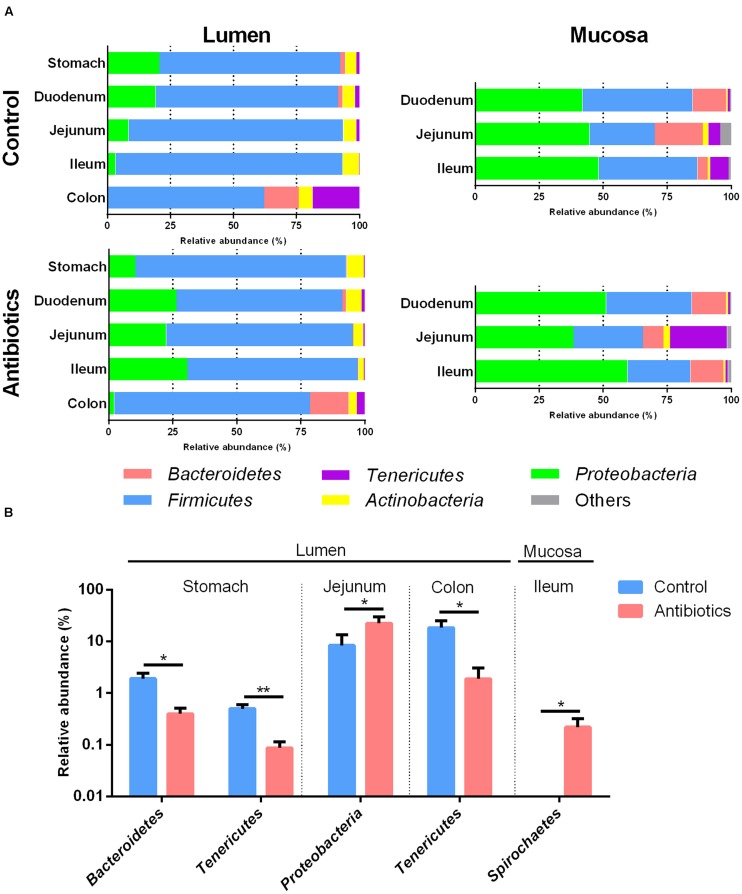
**(A)** The phylum-level taxonomic composition of gut bacterial communities at different gut locations in piglets of control and antibiotic group. **(B)** Effects of antibiotic treatment on the phylum level abundance of 16S rRNA gene sequences at different gut location. In **Figure 2B**, values are means ± SEMs (*n* = 8). Asterisks indicate different from control group (Student’s *t*-test): ^*^P < 0.05, ^*^^*^P < 0.01.

The bacterial composition varied in different anatomical regions in the piglet GIT. In the control group, *Firmicutes* (median, 69.48–94.65%) was most dominant in the lumen (**Figure 3A** and Supplementary Figure S3A), with *Streptococcus* (median 9.5–17.56%) and *Lactobacillus* (median, 10.27–12.88%) dominant in the stomach and small intestine and *Subdoligranulum* (median, 11.84%) dominant in the colon (Supplementary Table S2). *Proteobacteria* (median, 44.58–53.6%) was most dominant in the mucosa (**Figure 3A** and Supplementary Figure S3B), with *Escherichia-Shigella* (median, 11.65–16.12%), hereafter referred to *Escherichia*, dominant in the jejunum and ileum and *Alcaligenes* (median, 27.23%) dominant in the duodenum (Supplementary Table S3). The distribution of other dominant genera in the piglets not exposed to the antibiotic intervention is shown in Supplementary Tables S2, S3. For example, in the control group, the relative abundances of *Pseudomonas* and *Haemophilus* were higher in the lumen of the stomach and duodenum than in the jejunum, ileum, and colon (Supplementary Table S3). In addition, in the control group, the relative abundance of *Firmicutes* was significantly higher and the relative abundance of *Bacteroidetes* was lower in the lumen than those in the mucosa of the small intestine (Supplementary Figure S3C). These results indicated the spatial variation of gut microbiota between gut locations.

**FIGURE 4:**
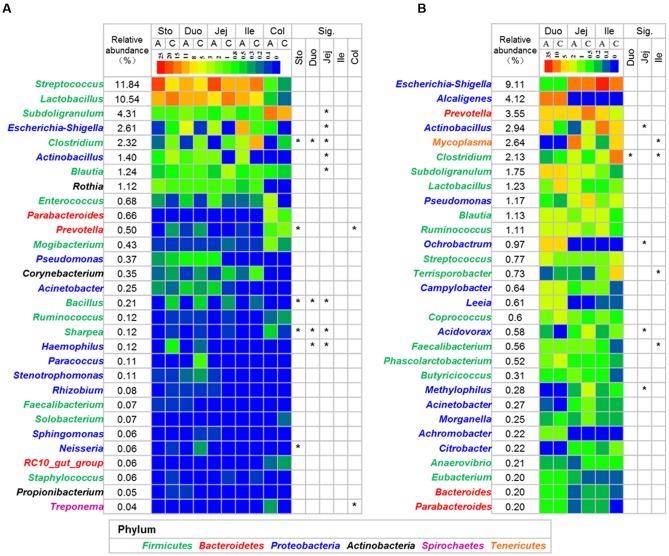
**Heatmap of the dominant genus in lumen (A)** and mucosa **(B)**. The mean relative abundance was calculated using all the samples in control and antibiotic group. Asterisks indicate different in the antibiotic group from the control group (Mann–Whitney *U-*test with and a false discovery rate < 5%). See Supplementary Tables S4, S5 for the complete dataset. Abbreviations: A, antibiotics; C, control; Col, colon; Duo, duodenum; Ile, ileum; Jej, jejunum; Sto, stomach.

The relative abundance of *Bacteroidetes* decreased in the lumen of the stomach in the antibiotic-treated piglets compared with the control group (*P* < 0.05, **Figure 3B**), with the relative abundance of *Prevotella* decreasing from 0.357 to 0.041% (*q* < 0.05, **Figure 4A** and Supplementary Table S4). The relative abundance of *Proteobacteria* increased significantly in the lumen of the jejunum (*P* < 0.05, **Figure 3B**), especially that of *Escherichia* and *Actinobacillus* genera (*q* < 0.05, **Figure 4A** and Supplementary Table S4) compared with the control group. The relative abundance of *Tenericutes* decreased in the lumen of the stomach and colon in the antibiotic-treated piglets compared with the control group (*P* < 0.05, **Figure 3B**). Among *Firmicutes*, the relative abundance of *Clostridium*, *Bacillus*, and *Sharpea* decreased in response to the antibiotic intervention in the lumen of the stomach, duodenum, and jejunum (*q* < 0.05, **Figure 4A**). In the colonic lumen, the antibiotic intervention significantly increased the relative abundance of *Treponema* and decreased the relative abundance of *Prevotella* compared with the control group (*q* < 0.05, **Figure 4A**). These results suggested that the effects of the antibiotics on the microbial composition in the lumen varied between gut locations.

The antibiotic intervention did not show effects on the relative abundance of dominant bacterial phyla in the mucosa at the end of the experiment but increased the relative abundance of the subdominant phylum *Spirochaeta* 44-fold in the mucosa of the ileum compared with the control group (*P* < 0.05, **Figure 3B**). Among *Proteobacteria*, the relative abundance of *Actinobacillus* decreased in response to the antibiotic intervention in the mucosa of the jejunum (*q* < 0.05, **Figure 4B** and Supplementary Table S5). Among *Firmicutes*, the relative abundance of *Faecalibacterium* increased and that of *Clostridium* decreased in response to the antibiotic intervention in the mucosa of the ileum (*q* < 0.05, **Figure 4B**). Among *Tenericutes*, the relative abundance of *Mycoplasma* decreased in response to the antibiotic intervention in the mucosa of the ileum (*q* < 0.05, **Figure 4B**). Collectively, these results indicated that the antibiotic intervention had profound effects on the lumen of the stomach and small intestine but few effects on the mucosa.

### Effect of the Antibiotic Intervention on the Representative Dominant OTUs in Piglets

Based on a 97% confidence level at the species level (OTU), the relative abundance of the major dominant phylotypes in the lumen and mucosa was identified (Supplementary Tables S6, S7). Among the top 50 dominant phylotypes in the lumen, the antibiotic intervention significantly altered the relative abundance of 12 OTUs in the stomach, 7 OTUs in the duodenum, 17 OTUs in the jejunum, 2 OTUs in the ileum, and 3 OTUs in the colon (Supplementary Figure S4). The relative abundance of *Clostridium disporicum* (OTU1212), *Eubacterium biforme* (OTU1865), and *L. mucosae* (OTU1088) decreased in the antibiotic intervention group in the lumen of the stomach, duodenum, and jejunum (*q* < 0.05). On the other hand, in response to the antibiotic intervention, the relative abundance of *S. porcorum* (OTU1762) and *S. suis* (OTU1070) increased in both the stomach and jejunum, and the relative abundance of *Escherichia coli* (OTU1800) increased in the jejunum (*q* < 0.05). The antibiotic intervention also reduced the relative abundance of *Gemmiger formicilis* (OTU218), *Ruminococcus lactaris* (OTU570), *R. faecis* (OTU592), and *Coprococcus comes* (OTU249; *q* < 0.05, Supplementary Figure S4). In the mucosa, the antibiotic intervention reduced the relative abundance of *L. mucosae* (OTU1088) in the duodenum but markedly increased the relative abundance of *S. suis* (OTU1070) in the ileum (Supplementary Figure S5). These results further confirmed the impact of the antibiotic intervention on the microbial composition at lower taxonomic levels.

### Effect of Antibiotics on Microbial Functional Capability

**FIGURE 5:**
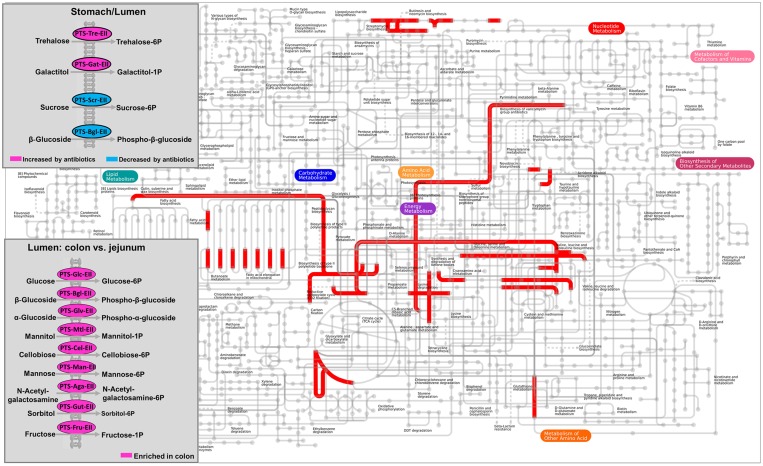
**Mapping the KEGG orthology groups (KOs) against KEGG modules based on the metagenomic prediction results.** In the overall metabolic map, pathways that are overrepresented in the mucosa versus lumen of duodenum were indicated in red. The carbohydrate transport phosphotransferase systems was highlighted in box. The significance of KOs selected was declared at FDR *q* < 0.05.

The metagenomic prediction showed that the functional capability of the lumen and mucosa differed (**Figure 5** and Supplementary Table S8). Mapping of KEGG IDs to iPath database showed that the colonic microbiota was enriched with functional potentials that are related to carbohydrate transport phosphotransferase systems, and that early-life antibiotics affected the phosphotransferase system and other metabolic pathways (**Figure 5**). The relative abundance of KOs predicted to be involved in glycosyltransferases, and starch and sucrose metabolism was higher in the lumen of both the duodenum and jejunum than in the mucosa (*q* < 0.05). The relative abundance of KOs predicted to be involved in propionate metabolism, butyrate metabolism, flagellar assembly, and lipopolysaccharide biosynthesis in the mucosa of the jejunum was higher than in the lumen (*q* < 0.05). These results indicated the different functional potential of the lumen versus the mucosa in the small intestine. Furthermore, the relative abundance of KOs predicted to be involved in the phosphotransferase system, including those that play a role in the phosphorylation of glucose, β-glucoside, and cellobiose, was higher in the lumen of the colon than in the jejunum (*q* < 0.05), as shown in **Figure 5**. These results suggested that the functional potential of the gut microbiota varied according to gut locations.

The metagenomic prediction also showed that compared to the control group, the antibiotic intervention increased the relative abundance of KOs predicted to be involved in the phosphorylation of trehalose and galactitol, whereas it decreased those involved in the phosphorylation of sucrose and β-glucoside in the lumen of the stomach (*q* < 0.05, **Figure 5**). We further analyzed the KOs predicted to be involved in carbohydrate-active enzymes. The impact of the antibiotic intervention on the enzymes varied among the gut locations (Supplementary Table S9). For example, in the lumen of the jejunum, the antibiotic intervention increased the relative abundance of KOs belonging to glycoside hydrolases (xylan 1, 4-beta-xylosidase) and glycosyltransferases (trehalose-phosphatase) compared with the control group. In addition, the antibiotic intervention affected KOs associated with antibiotic and ion resistance (**Table 2**). In the lumen of the colon, the antibiotic intervention increased the relative abundance of KO-related genes *emrK* and *emrY* that confer resistance to tetracycline more than 70-fold compared with the control group (*q* < 0.05). These results showed that antibiotics may have profound effects on KOs associated with antibiotic resistance in the lumen but not the mucosa.

**Table 2:** Effect of antibiotics on the antibiotic resistance genes and ion resistance genes predicted to be represented in the gut microbiota by metagenomic prediction.

Description	Confer resistance to	Gene	Fold change(Antibiotics vs. Control)	Gut location
**FDR *P* < 0.05**				
Small multidrug resistance protein, SMR family	Aminoglycoside, β-lactam	*emrE*	32.1	Jejunum/Lumen
Multidrug resistance protein A	Fluoroquinolone	*emrA*	2.74	Colon/Lumen
MFS transporter, FSR family, fosmidomycin resistance protein	Fosmidomycin	*fsr*	1.92	Colon/Lumen
Two-component system, OmpR family, copper resistance phosphate regulon response regulator	Copper	*cusR*	1.93	Colon/Lumen
MFS transporter, DHA2 family, multidrug resistance protein	Aminocoumarin (for example, Novobiocin)^a^	*yebQ*	3.26	Colon/Lumen
Two-component system, OmpR family, bacitracin resistance sensor histidine kinase	Bacitracin	*bceS*	0.38	Duodenum/Lumen
Two-component system, OmpR family, bacitracin resistance response regulator BceR	Bacitracin	*bceR*	0.38	Duodenum/Lumen
MFS transporter, ACDE family, multidrug resistance protein	Cationic antimicrobial peptides^a^	*yitG*	0.27 0.17	Stomach/Lumen Duodenum/Lumen
**Unadjusted *P* > 0.05**				
Multidrug resistance protein A	Fluoroquinolone	*emrA*	38.9	Jejunum/Lumen
Multidrug resistance protein K	Tetracycline	*emrK*	81.2 70.1	Duodenum/Lumen Colon/Lumen
MFS transporter, multidrug resistance protein Y	Tetracycline	*emrY*	81.2 70.1	Duodenum/Lumen Colon/Lumen
MFS transporter, DHA1 family, multidrug resistance protein	Chloramphenicol	*mdtL*	58.3 70.1	Duodenum/Lumen Colon/Lumen
zinc resistance-associated protein	Zinc	*zraP*	40.6	Colon/Lumen
Multiple antibiotic resistance protein	Tetracycline	*marB*	34.8	Colon/Lumen
AraC family transcriptional regulator, multiple antibiotic resistance protein	Tetracycline, chloramphenicol, fluoroquinolone	*marA*	34.9	Colon/Lumen
MFS transporter, DHA1 family, multidrug/chloramphenicol efflux transport protein	Tetracycline, puromycin, chloramphenicol, erythromycin, certain Aminoglycosides and Fluoroquinolones	*mdfA*	31.7	Colon/Lumen
Macrolide-specific efflux protein	Macrolide	*macA*	9.30	Colon/Lumen

*^a^According to search by Resistance Gene Identifier in the Comprehensive Antibiotic Resistance Database (http://arpcard.mcmaster.ca) (McArthur et al., 2013) and the resistance phenotype information in Interpro database (http://www.ebi.ac.uk/interpro/).*

**FIGURE 6:**
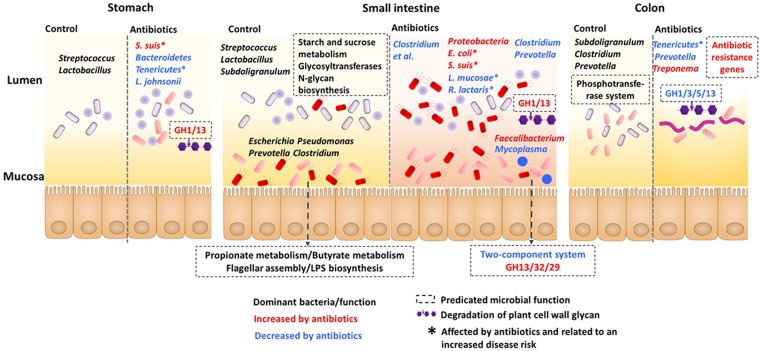
**Proposed function model of the impact of antibiotics on the microbiota and the predicted microbiota function along the stomach and intestine**.

To summarize the effects of the early-life in-feed antibiotics on gut microbiota in piglets, a function model was proposed as shown in **Figure 6**. Overall, the microbial communities and functions differed in gut locations, especially in the lumen and mucosa of the small intestine. Antibiotics induced differential and compartmentalized alterations in gut microbiota, with marked changes in the foregut versus hindgut and in lumen versus mucosa.

## Discussion

In this study, by high-throughput sequencing of 16S rRNA genes, we analyzed the impact of an early-life antibiotic intervention on microbial community diversity and functions in the GIT of piglets. The results revealed that the antibiotic intervention induced a differential and compartmentalized alteration in the gut microbiota.

### Spatial Variations in the Microbiota of the Stomach versus the Intestine and Lumen versus the Mucosa

The study revealed large spatial variation in the microbial composition of the GIT of the piglets, and found that although not as diverse as in the colon, the stomach and duodenum had a highly diverse microbiota. The microbiota of gastric and duodenal lumen samples from the piglets showed greater diversity than microbiota of ileal digesta samples. A similar result was observed in a mice study (Gu et al., 2013). Allochthonous bacteria introduced from external environments can pass through the stomach and duodenum but may not reach the jejunum and ileum due to intestine-specific environmental condition (i.e., pH and nutrient gradient). In the present study, the relative abundances of *Escherichia*, *Pseudomonas*, and *Haemophilus* were high in the lumen of the stomach and duodenum, but reduced in the lumen of the jejunum and ileum. *Escherichia*, *Pseudomonas*, and *Haemophilus* spp. are generally considered as opportunistic pathogens (Ezepchuk, 2013). The difference in oxygen gradient and nutrient substrate along the lumen of the small intestine may have affected the microbial distribution. The results suggest the importance of the spatial variation between the stomach and intestine, which may partly explain the development or succession of microbiota in the intestine (during antibiotic treatments or environmental stress).

In addition to the microbiota variation in longitudinal locations, in the radial locations, the microbial communities of the gut lumen and mucosa were markedly different. Facultative anaerobes, such as *Escherichia*, dominated in the mucosa of the small intestine, whereas its abundance decreased in the lumen. A similar distribution was observed in the gut of mice (Gu et al., 2013). A previous study showed that environmental heterogeneity, such as different oxygen exposure, pH, and substrate availability, appeared to affect the microbial distribution in the gut (Hao and Lee, 2004). The intestinal oxygen content decreased from the mucosa to lumen in the small intestine (Donaldson et al., 2016). The aforementioned findings explain the location-specific distribution of the gut microbiota of the piglets in the present study.

In the present study, metagenomic prediction suggested that the gut microbiota of lumen and mucosa differed in functional capability of immunological regulation. Compared to the lumen, Gram-negative *Proteobacteria* dominated in the mucosa, and this was accompanied by increased KOs involved in flagellar assembly and lipopolysaccharide biosynthesis. Bacteria within *Proteobacteria*, such as *Escherichia*, can produce lipopolysaccharides and flagella, which serve as microbe-associated molecular patterns that are recognized by Toll-like receptors 4 and 5, respectively (Mu et al., 2015). The signals triggered by flagella are essential in regulating mucosal immune development (Cullender et al., 2013). The findings of the present study point to the possibly different roles of mucosal and luminal bacteria in regulating epithelial health.

In longitudinal locations, the microbiota differed in the functional potential of phosphotransferase system along stomach and colon. The microbiota in the colonic lumen was predicted to code more genes involved in the phosphotransferase system than those in the jejunum, including enzymes phosphorylating cellobiose. Metagenomic sequencing of the microbiome in the gut of growing pigs suggested that the microbiome in the ileum lumen lacked cellobiose phosphorylase homologs compared to the cecum and colon (Looft et al., 2014a). Therefore, these results suggest a functional difference in the phosphotransferase system exists along the gut in both piglets and growing pigs.

### Differential and Compartmentalized Alterations in Gut Microbiota in Response to the Early-Life Antibiotic Intervention

In the present study, both the microbiota analysis and metagenomic prediction showed compartmentalized alterations in the gut microbiota in response to the early-life antibiotics, with the impacts varying on each gut locations. Early-life in-feed antibiotic predominantly shifts microbiota in the foregut rather than hindgut of piglets.

The compartmentalized alteration was evidenced by the fact that no change was consistently occurred across the stomach to the colon and from lumen to mucosa. In the lumen, the effect of antibiotics was dramatic in the foregut (stomach, duodenum, and jejunum), but then weakened gradually, with largest effect in the jejunum and less effects in the colon as revealed by the numbers of genera affected. This may be possibly related to the gradual dilution of antibiotics in the gut, which led to the weakened effects. Pharmacokinetic analysis of olaquindox (Yang B. et al., 2014), oxytetracycline (Mevius et al., 1986), kitasamycin (Sun et al., 2010) showed that all these antibiotics could be absorbed by pig gut after oral administration. The abundances of bacteria such as *Clostridium*, *Bacillus*, and *Sharpea* were consistently decreased in the lumen of stomach, duodenum, and jejunum. These bacteria groups may respond similarly to the early-life antibiotics. In the mucosa of small intestine, however, the microbial composition showed a strikingly compartmentalized variation after the antibiotic treatment in the present study, which may be related to the location-specific compositions along the mucosa. Different locations in the mucosa may differ in niche specificity and nutrient availability. The mucus layer in the small intestine is discontinuously secreted and therefore nutrient source for microbes differs along the mucosa (Brown et al., 2013). In addition, some bacteria, such as *S. suis*, are specifically predominant in the lumen of stomach and small intestine in the weaning piglets, but not in mucosa (Su et al., 2008). Therefore, it could be expected that the microbial distribution may account for the compartmentalized alterations in gut microbiota after the early-life antibiotics. Collectively, our findings showed an early-life antibiotic-induced dramatic and location-specific alteration in gut microbiota in the stomach and intestine. Given that the present study monitored the microbial composition after antibiotic treatment for 5 weeks, some transient effects of antibiotics may be missed, which can be studied in future investigations

The early-life antibiotics increased the abundance of potential pathogens and decreased those with beneficial function. Among the bacteria groups affected, a number of the bacterial species have previously been correlated with disease risk in humans or pigs. For example, the decreased relative abundances of phylotypes *L. mucosae*, *R. lactaris*, and *C. comes* and increased relative abundances of the phylum *Proteobacteria*, genus *Escherichia*, and phylotypes *S. suis* and *S. pasteurianus* have been associated with disease condition. *L. mucosae* is regarded as a beneficial microbe and is decreased in humans with Crohn’s disease (Neut et al., 2002). Decreased abundance of *R. lactaris* was reported in the feces of patients with cystic fibrosis (Duytschaever et al., 2013). An increase in *Escherichia* was observed in feces of piglets with post-weaning diarrhea (McAllister et al., 1979). Meanwhile, increases of *Escherichia* have been found to be a side effect of some antibiotic treatments, independent of its pathogenicity status (Looft et al., 2012; Stecher et al., 2013). Thus, whether the increase of *Escherichia* after early-life antibiotic intervention affects host immune status may be interesting for further research. In pigs, *S. suis* is an important cause of a wide variety of infections, including meningitis, pneumonia, septicemia, and arthritis (Zhang et al., 2015). In addition, the present study found that the early-life antibiotics decreased the relative abundance of *Tenericutes* in the lumen of the stomach and colon. Previous research reported that a decrease in the abundance of *Tenericutes* was associated with exacerbated asthma in vancomycin-treated mice (Russell et al., 2012). Collectively, these results point to a potentially detrimental impact of early-life antibiotic intervention on the composition of gut microbiota in piglets.

Interestingly, we found that the gut location explained more of the variation in microbiota composition than antibiotics in the present study, as revealed in RDA analysis. Similar finding was also observed for microbial communities in the ileum, cecum, and colon of growing pigs with in-feed antibiotics (mixture of chlortetracycline, sulfamethazine, and penicillin) (Looft et al., 2014a). Both intestinal physiological conditions and environmental factors affect the microbial communities. Normally, gut microbiota forms a stable colonization that is positively selected by nutrient availability, immune system, and other factor, such as oxygen gradient (Lozupone et al., 2012; Donaldson et al., 2016). Although exposed to the antibiotic perturbation at early life, the ecological environment in the gut may not be changed to a large extent. Thus, antibiotic intervention is a minor variable compared to the gut location. In addition, in the present study, as in-feed antibiotics, low dosage was used, which may lead to the antibiotic effect not great enough to induce a huge change in gut microbiota in piglets and overcome the impact of gut location on the microbiota.

In addition, another interesting finding of the present study is that the antibiotic intervention seemed to increase KOs predicted to be involved in antibiotic resistance genes (ARGs). These increases occurred mainly in the colonic lumen but not in the stomach, duodenum, or jejunum. In growing pigs, in-feed antibiotics using a chlortetracycline, sulfamethazine, and penicillin mixture were reported to augment the abundance of the *mdtL* gene, which confers resistance to chloramphenicol (Looft et al., 2012, 2014b), and that of the *emrE* gene, which confers resistance to aminoglycoside (Looft et al., 2014b), in the large intestine. Consistent with these results, in the present study, the PICRUSt prediction also found increased abundance of *emrE* and *mdtL* genes in the colonic lumen of piglets. It should be noted that ARGs can be horizontally transferred. When considered in conjunction with the studies by Looft et al. (2012, 2014b), the present findings suggest that colonic microbiota may possibly be a key reservoir of ARGs.

Based on the PICRUSt prediction, the present study revealed alterations in the abundance of potential genes involved in antibiotic resistance. However, the prediction algorithm depended on representative genomes from the sequenced bacteria. Other types of investigations, for example, studies using a metagenomics sequencing-based approach, may allow to determine the function of porcine gut microbiota, which can be studied in future research. Nevertheless, the metagenomic prediction in the present study also provides valuable insights into the effect of antibiotic interventions in early life on microbiota function in piglets.

## Conclusion

This study simultaneously investigated the spatial variation of gut microbiota and its response to an early-life antibiotics intervention. It is striking that the early-life antibiotic intervention resulted in differential and compartmentalized alterations in the gut microbiota from the stomach to colon. The diversity of the microbiota in the stomach and duodenum was highly similar, but markedly different to that in the jejunum and ileum. The dominant bacteria were similar in the stomach and small intestine, but different to those in the colon. The composition and functional potential of the microbiota in the mucosa differed from those in the lumen. The early-life antibiotic intervention caused a more marked alteration in the microbial composition and functional potential in the foregut versus hindgut, pointing to an antibiotic-induced differential and compartmentalized impact. The antibiotic intervention led to an increase in bacteria groups associated with an increased risk of disease. Furthermore, the increase in potential ARGs may point to collateral antibiotic-induced alterations in the gut microbiota of the piglets. The marked compartmentalized alterations induced by the early-life antibiotics highlights the importance of considering the foregut microbiota in antibiotic-related research. This study provides new insights into the membership of the microbiota along the GIT of piglets and highlights the importance of considering the foregut microbiota in health management of piglets.

## Author Contributions

CM and YY performed the experiments. CM, YY, and YS analyzed the data. CM, YY, and WZ wrote the paper. YS, EZ, and WZ contributed the conception of the work. EZ and WZ revised it critically for important content. WZ had primary responsibility for the final content.

## Conflict of Interest Statement

The authors declare that the research was conducted in the absence of any commercial or financial relationships that could be construed as a potential conflict of interest.

## Footnotes

1 http://arpcard.mcmaster.ca

2 http://www.cazy.org/
