# Supplementary material for: Differences in Microbiota Membership along the Gastrointestinal Tract of Piglets and Their Differential Alterations Following an Early-Life Antibiotic Intervention
Source: Front Microbiol. 2017 May 9;8:797. doi: 10.3389/fmicb.2017.00797 (PMC5422473; doi:10.3389/fmicb.2017.00797)
Supplement: Supplementary file 1 [file Presentation_1.pdf]

Differences in Microbiota Membership along the Gastrointestinal Tract of Piglets and their Differential Alterations Following an Early-Life Antibiotic Intervention

Chunlong Mu[1], Yuxiang Yang[1], Yong Su[1], Erwin G. Zoetendal[1,2], Weiyun Zhu[1]*

[1]Laboratory of Gastrointestinal Microbiology, College of Animal Science and Technology, Nanjing Agricultural University, Nanjing, China

[2]Laboratory of Microbiology, Wageningen University, Wageningen, The Netherlands

Supplementary material

Summary

The supporting information includes five supplementary figures and nine supplementary tables.

Figure S1. Principle coordinate analysis plot

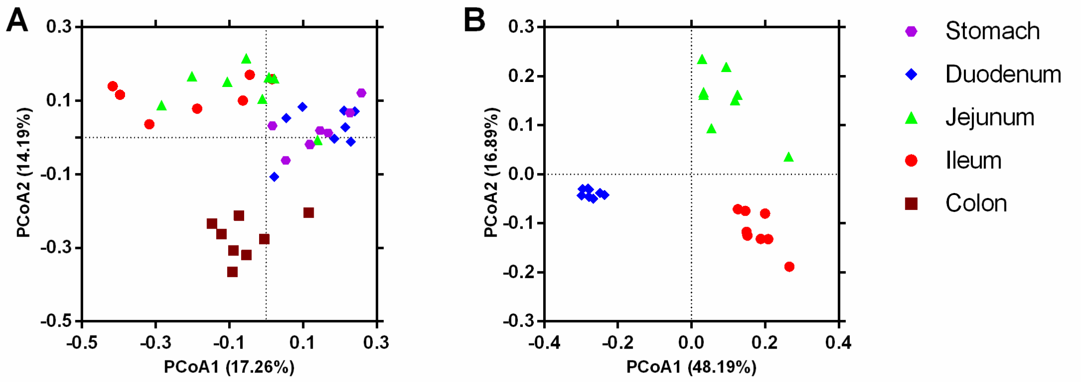

Principle coordinate analysis of all samples including luminal samples in control group (**A**) and mucosal samples in control group (**B**) by unweighted Unifrac distance.

Figure S2. Alpha-diversity index plot

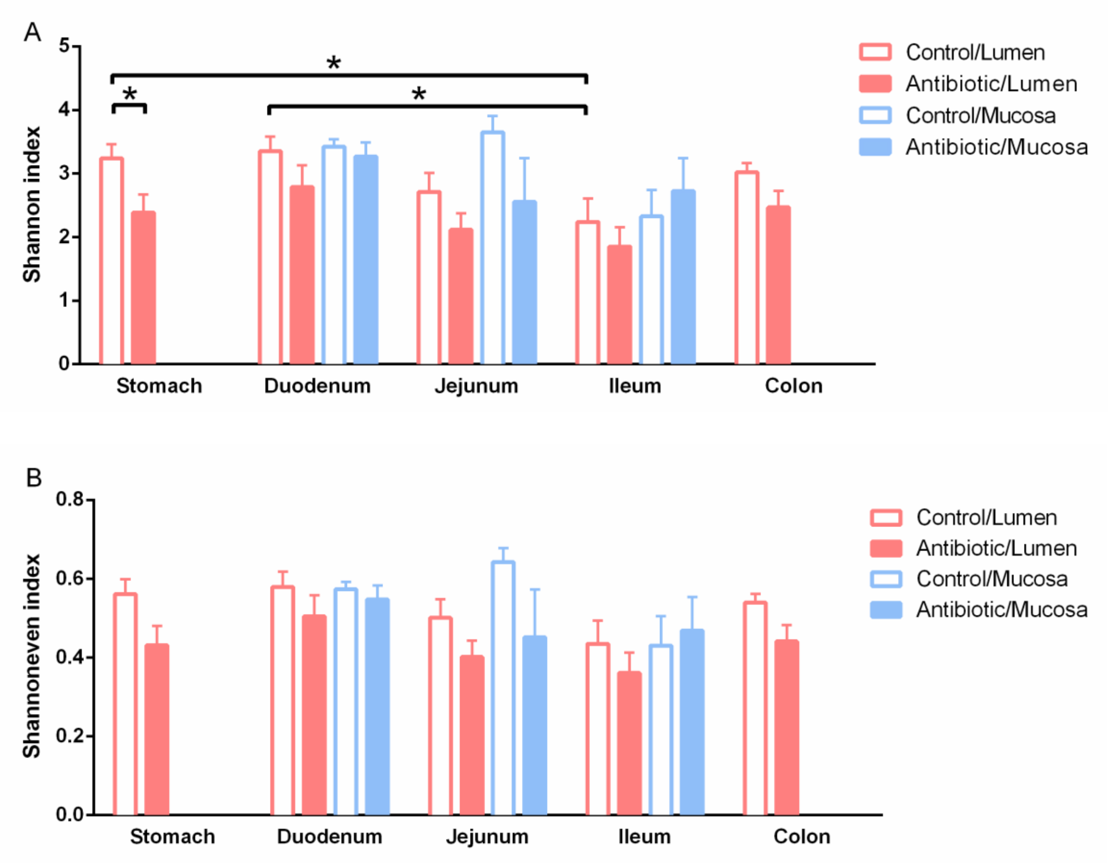

Shannon (**A**) and Shannoneven (**B**) index of gut microbiota across stomach and colon. Data were expressed as mean ± SEM, n = 8 for each group. Asterisks indicate different from between groups (Student's t-test, *P < 0.05).

Figure S3. Relative abundance of dominant phylum

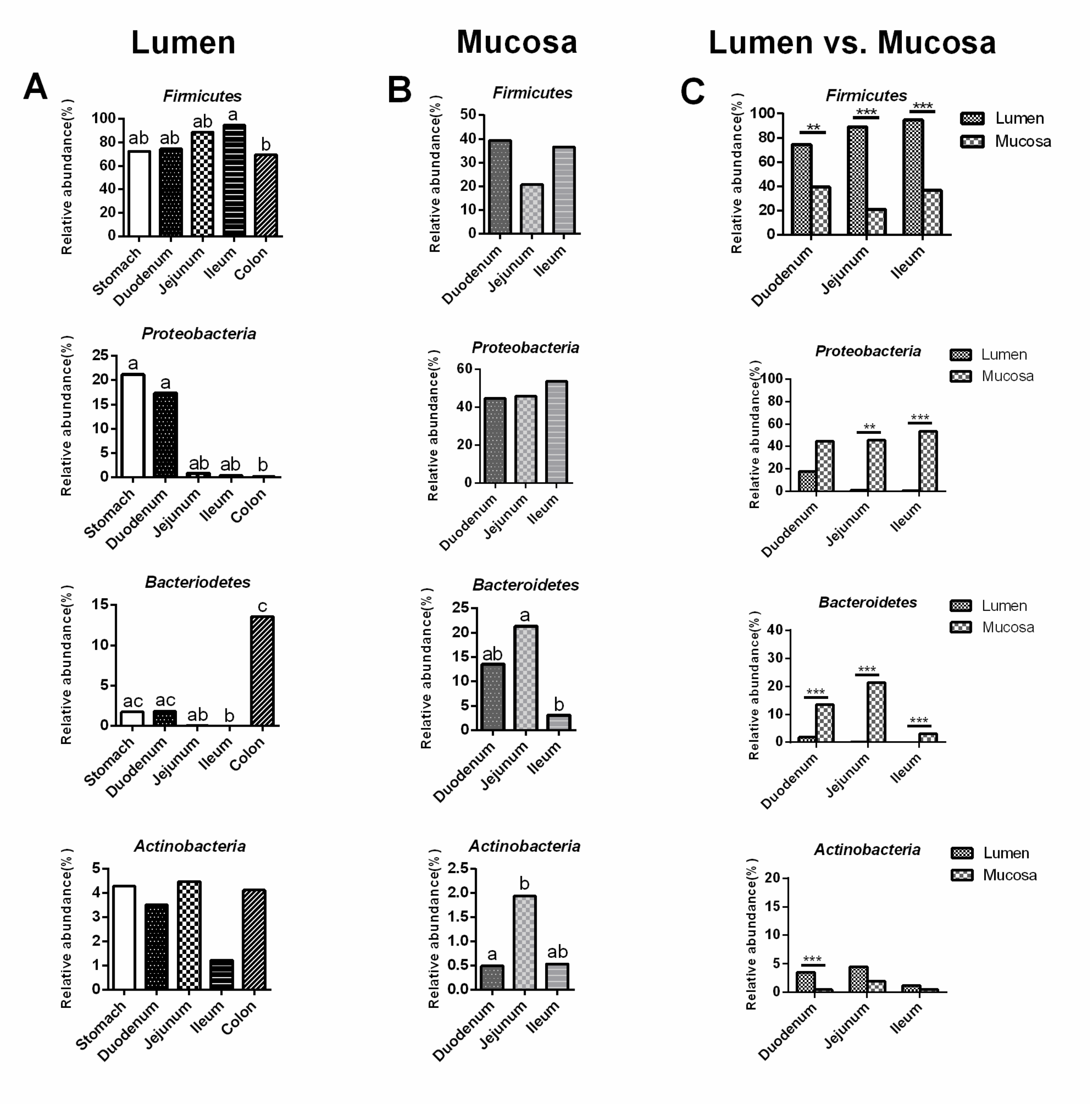

Relative abundance of dominant phylum in lumen (**A**), mucosa (**B**), and difference between lumen and mucosa (**C**). For (**A**) and (**B**), values are medians (*n* = 8). Different letters indicate significant difference based on non-parametric Kruskal–Wallis ANOVA. For (**C**), values are medians (*n* = 8). Asterisks indicate different in lumen compared with mucosa (Mann-Whitney *U*-test): **P < 0.01; *** P < 0.001.

Figure S4. Heatmap of top 50 phylotypes in the lumen

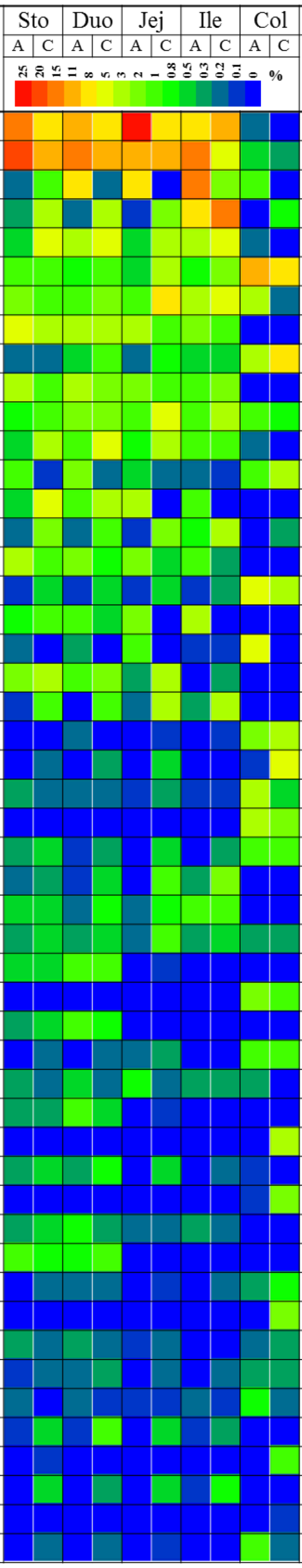

OTU		Relative abundance (%)	Significance Sto	Duo	Jej	Ile	Col
OTU1762	*Streptococcus porcorum*	13.0			*		
OTU16	*Lactobacillus reuteri*	12.6					
OTU1800	*Escherichia fergusonii*	5.9			*		
OTU1212	*Clostridium disporicum*	4.4	*	*	*		
OTU1757	*Lactobacillus johnsonii*	3.9	*				
OTU218	*Gemmiger formicilis*	3.8			*		
OTU295	*Streptococcus pasteurianus*	3.4					
OTU1174	*Streptococcus porcorum*	3.3					
OTU478	*Gemmiger formicilis*	2.9			*		
OTU885	*Rothia nasimurium*	2.2					
OTU446	*Blautia wexlerae*	2.2					
OTU1392	*Lactobacillus acidophilus*	2.0					
OTU937	*Gemmiger formicilis*	2.0					
OTU987	*Actinobacillus indolicus*	1.7			*		
OTU1751	*Terrisporobacter mayombei*	1.4	*	*			*
OTU1070	*Streptococcus suis*	1.4	*		*		
OTU1865	*Eubacterium biforme*	1.4	*	*	*		
OTU554	*Actinobacillus porcinus*	1.1					
OTU1618	*Enterococcus cecorum*	1.1					
OTU188	*Rhodoligotrophos appendicifer*	1.1					
OTU835	*Streptococcus pasteurianus*	1.0			*		
OTU73	*Eubacterium contortum*	0.9			*		
OTU979	*Dielma fastidiosa*	0.9	*		*	*	
OTU1890	*Mogibacterium neglectum*	0.8					
OTU1643	*Parabacteroides distasonis*	0.7					
OTU570	*Ruminococcus lactaris*	0.7			*		
OTU1607	*Corynebacterium casei*	0.6					
OTU242	*Streptococcus ferus*	0.5		*			
OTU592	*Ruminococcus faecis*	0.5			*		
OTU1551	*Citrobacter werkmanii*	0.5					
OTU776	*Parabacteroides distasonis*	0.5					
OTU24	*Pseudomonas monteilii*	0.5					
OTU648	*Ruminococcus faecis*	0.4					
OTU1570	*Enterococcus hirae*	0.4					
OTU1325	*Acinetobacter johnsonii*	0.4					
OTU257	*Anaerobacterium chartisolvens*	0.4					*
OTU249	*Coprococcus comes*	0.4			*		
OTU192	*Spiroplasma eriocheiris*	0.3					*
OTU9	*Lactobacillus delbrueckii*	0.3					
OTU810	*Veillonella caviae*	0.3					
OTU1188	*Collinsella aerofaciens*	0.3	*				
OTU423	*Spiroplasma alleghenense*	0.3	*	*			
OTU1388	*Streptococcus pleomorphus*	0.3					
OTU767	*Olsenella umbonata*	0.3			*	*	
OTU252	*Eubacterium cylindroides*	0.3					
OTU384	*Bacillus atrophaeus*	0.3	*				
OTU499	*Eubacterium coprostanoligenes*	0.3					
OTU1088	*Lactobacillus mucosae*	0.2	*	*	*		
OTU734	*Enorma massiliensis*	0.2	*				
OTU989	*Sharpea azabuensis*	0.2	*	*	*		

Asterisks indicate significant difference between control and antibiotic treatment (Mann-Whitney *U*-test $P < 0.05$).The mean relative abundance was calculated using all the samples in control and antibiotic group. A, antibiotics; C, control; Col, colon; Duo, duodenum; Ile, ileum; Jej, jejunum; Sto, stomach.

Figure S5. Heatmap of top 50 phylotypes in the mucosa

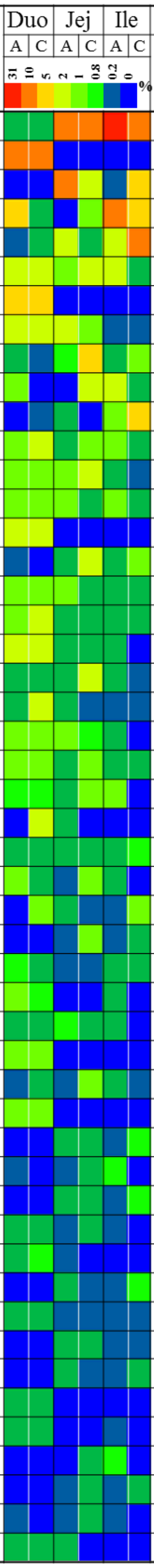

OTU		Relative abundance (%)	Sig. Duo	Sig. Jej	Sig. Ile
OTU1800	*Escherichia fergusonii*	8.5			
OTU491	*Alcaligenes aquatilis*	3.9			
OTU1680	*Mycoplasma sualvi*	2.5			*
OTU987	*Actinobacillus indolicus*	2.0			
OTU1212	*Clostridium disporicum*	1.8	*		*
OTU446	*Blautia wexlerae*	0.9			
OTU627	*Ochrobactrum pseudogrignonense*	0.9		*	
OTU218	*Gemmiger formicilis*	0.9			
OTU1085	*Pseudomonas azotoformans*	0.8		*	
OTU554	*Actinobacillus porcinus*	0.7		*	
OTU1751	*Terrisporobacter mayombei*	0.7			*
OTU959	*Prevotella paludivivens*	0.6			
OTU943	*Prevotella copri*	0.6			
OTU570	*Ruminococcus lactaris*	0.6			
OTU270	*Pandoraea thiooxydans*	0.6			
OTU764	*Acidovorax soli*	0.5		*	
OTU249	*Coprococcus comes*	0.5			
OTU1458	*Phascolarctobacterium succinatutens*	0.5			
OTU478	*Gemmiger formicilis*	0.5			
OTU263	*Prevotella copri*	0.5			
OTU16	*Lactobacillus reuteri*	0.5			
OTU256	*Faecalibacterium prausnitzii*	0.4			*
OTU2016	*Prevotella copri*	0.4			
OTU1449	*Campylobacter hyointestinalis*	0.4			
OTU1392	*Lactobacillus acidophilus*	0.3			
OTU295	*Streptococcus pasteurianus*	0.3			
OTU937	*Gemmiger formicilis*	0.3			*
OTU1757	*Lactobacillus johnsonii*	0.3			
OTU1258	*Methylophilus luteus*	0.3		*	
OTU1762	*Streptococcus porcorum*	0.3			
OTU1920	*Prevotella oralis*	0.2			
OTU2005	*Butyricicoccus pullicaecorum*	0.2			
OTU1756	*Campylobacter lanienae*	0.2			
OTU1228	*Prevotella oris*	0.2			
OTU1150	*Achromobacter mucicolens*	0.2			
OTU1551	*Citrobacter werkmanii*	0.2			
OTU784	*Prevotella oralis*	0.2			
OTU24	*Pseudomonas monteilii*	0.2			
OTU592	*Ruminococcus faecis*	0.2			
OTU1865	*Eubacterium biforme*	0.2			
OTU1396	*Brevundimonas naejangsanensis*	0.2			
OTU1323	*Pseudoflavonifractor capillosus*	0.2			
OTU1325	*Acinetobacter johnsonii*	0.2			
OTU71	*Klebsiella pneumoniae*	0.1			
OTU29	*Sporobacter termitidis*	0.1			
OTU526	*Bacteroides stercoris*	0.1			
OTU746	*Barnesiella intestinihominis*	0.1			*
OTU981	*Brucella ceti*	0.1			
OTU1240	*Intestinibacter bartlettii*	0.1			
OTU648	*Ruminococcus faecis*	0.1			

Asterisks indicate significant difference between control and antibiotic treatment (Mann-Whitney *U*-test $P < 0.05$). The mean relative abundance was calculated using all the samples in control and antibiotic group. A, antibiotics; C, control; Col, colon; Duo, duodenum; Ile, ileum; Jej, jejunum; Sto, stomach.

Table S1. The composition and ingredient of experimental diets

Items	Content
Ingredients (%)	
corn	40
rice, broken	15
soybean meal, fermented	10
soybean meal, de-hulled	6
spray dried animal plasma	5
whey powder	7
fish meal	4
sugar	4.5
glucose	3
soybean oil	1.5
L-Lysine-HCl (98%)	0.3
L-Methionine	0.15
L-Threonine	0.2
L-Tryptophan	0.05
L-Isoleucine	0.05
L-Valine	0.05
Salt	0.3
Limestone	1.1
CaHPO4	0.8
Vitamin mixture[1]	0.2
Mineral mixture[2]	0.8
Total	100
Nutrient composition, %	
Crude protein	20.2
Digestible energy (Mcal/kg)	3.4
Total calcium	0.85
Total phosphorus	0.7
Digestible Lys	1.45
Digestible Met+Cys	0.79
Digestible Thr	0.81
Digestible Trp	0.23
Digestible Ile	0.74
Digestible Leu	1.45
Digestible Val	0.89

[1] Vitamin mixture supplied the following per kg complete diet: vitamin A, 15,000 IU; vitamin D3, 3,000 IU; vitamin E, 150 mg; vitamin K3, 3 mg; vitamin B1, 3 mg; vitamin B2, 6 mg; vitamin B6, 5 mg; vitamin B12, 0.03 mg; niacin, 45 mg; vitamin C, 250 mg; calcium pantothenate, 9 mg; folic acid, 1 mg; biotin, 0.3 mg; choline chloride, 500 mg.

[2] Mineral mixture supplied the following per kg complete diet: Fe, 170 mg; Cu, 150 mg; I, 0.90 mg; Se,0.2 mg; Zn, 150 mg; Mg, 68 mg; Mn, 80 mg; Co, 0.3 mg.

Table S2. Abundance of the dominant genus in the lumen of stomach, duodenum, jejunum, ileum and colon. The data of relative abundance (%) are expressed as median. Different letters indicate significant difference based on non-parametric Kruskal–Wallis ANOVA ($P < 0.05$), followed by multiple comparisons of mean ranks.

Items	Stomach	Duodenum	Jejunum	Ileum	Colon
Streptococcus	9.50a	10.64a	15.23a	17.56a	0.17b
Lactobacillus	12.88a	10.27a	11.90a	10.42ab	0.14b
Subdoligranulum	0.85a	1.57ab	2.72ab	0.51a	11.84b
Escherichia-Shigella	0.22a	0.16ab	0.01bc	0.03abc	0.01c
Clostridium	1.02a	0.88a	0.18a	0.39a	0.27a
Actinobacillus	1.01a	0.26ab	0.00b	0.00b	0.00b
Blautia	0.64ab	0.95ab	2.82a	0.90ab	0.11b
Rothia	0.91a	0.96a	0.53a	0.10ab	0.00b
Enterococcus	0.10a	0.17a	0.05ab	0.18a	0.00b
Parabacteroides	0.01ab	0.01ab	0.00a	0.00a	0.33b
Prevotella	0.36ac	0.18abc	0.00ab	0.00b	1.87c
Mogibacterium	0.08a	0.05a	0.19a	0.01a	0.11a
Pseudomonas	0.61a	0.45a	0.01ab	0.00b	0.00b
Corynebacterium	0.20a	0.20ab	0.15ab	0.27ab	0.02b
Acinetobacter	0.30a	0.37a	0.01ab	0.00b	0.00b
Bacillus	0.37a	0.76a	0.14ab	0.04ab	0.01b
Ruminococcus	0.05ab	0.14ab	0.04ab	0.03a	0.22b
Sharpea	0.05a	0.05a	0.04a	0.02a	0.00a
Haemophilus	0.21a	0.12a	0.00b	0.00b	0.00b
Paracoccus	0.02ac	0.07a	0.00bc	0.00bc	0.00b
Stenotrophomonas	0.14a	0.17a	0.01ab	0.00b	0.00b
Rhizobium	0.12a	0.10a	0.01ab	0.00ab	0.00b
Faecalibacterium	0.10a	0.07a	0.01ab	0.00b	0.02ab
Solobacterium	0.05a	0.03a	0.03a	0.02a	0.11a
Sphingomonas	0.03a	0.05a	0.07a	0.00a	0.00a
Neisseria	0.04a	0.02ab	0.00ab	0.00ab	0.00b
RC10_gut_group	0.00ab	0.01ab	0.00a	0.00a	0.09b
Staphylococcus	0.05a	0.07a	0.06a	0.00ab	0.00b
Propionibacterium	0.04a	0.03a	0.00b	0.00b	0.00b
Treponema	0.00a	0.00a	0.00a	0.00a	0.00a

Table S3. Abundance of the dominant genus in the mucosa of duodenum, jejunum, and ileum. The data of relative abundance (%) are expressed as median. Different letters indicate significant difference based on non-parametric Kruskal–Wallis ANOVA ($P < 0.05$), followed by multiple comparisons of mean ranks.

Items	Duodenum	Jejunum	Ileum
Escherichia-Shigella	0.24a	11.65b	16.12b
Alcaligenes	27.23a	0.01b	0.00b
Prevotella	8.03ab	9.58a	0.85b
Clostridium	0.56a	0.50a	9.41b
Lactobacillus	1.32a	0.30a	1.32a
Actinobacillus	0.31a	0.78a	0.44a
Mycoplasma	0.01a	0.63a	0.78a
Pseudomonas	0.29a	5.40b	1.45ab
Subdoligranulum	7.49a	2.43ab	0.38b
Terrisporobacter	0.21ab	0.06a	2.55b
Blautia	2.58a	1.13ab	0.30b
Ruminococcus	2.71a	1.56ab	0.31b
Ochrobactrum	6.84a	0.01b	0.00b
Acidovorax	0.04a	3.29b	0.52ab
Streptococcus	1.14a	1.11a	1.40a
Leeia	2.32a	0.02ab	0.00b
Campylobacter	0.18a	0.18a	0.02a
Coprococcus	1.66a	0.76ab	0.32b
Phascolarctobacterium	1.39a	0.31ab	0.03b
Faecalibacterium	1.52a	0.82ab	0.11b
Methylophilus	0.02a	1.31b	0.35ab
Morganella	0.49a	0.48a	0.01a
Acinetobacter	0.03a	1.08b	0.26ab
Citrobacter	0.03a	0.77b	0.23ab
Anaerovibrio	0.29a	0.49a	0.05a
Achromobacter	1.62a	0.02b	0.02b
Butyricicoccus	0.26a	0.49a	0.09a
Parabacteroides	0.65a	0.37ab	0.01b
Bacteroides	0.49a	0.20ab	0.05b
Eubacterium	0.69a	0.10b	0.08b

Table S4. Genus-level effects of antibiotic treatment (Ant) on the dominant bacteria in the lumen compared with the control group (Con). The data of relative abundance (%) are expressed as median. Asterisks indicate different from control group (Mann-Whitney *U*-test and a false discovery rate < 5%).

Items	Stomach		Duodenum		Jejunum		Ileum		Colon	
	Con	Ant	Con	Ant	Con	Ant	Con	Ant	Con	Ant
Streptococcus	9.498	21.224	10.639	10.245	15.229	16.905	17.560	10.611	0.174	0.156
Lactobacillus	12.878	8.810	10.274	11.459	11.898	2.812	10.416	11.321	0.139	0.034
Subdoligranulum	0.855	0.396	1.566	1.512	2.716	0.389*	0.515	0.174	11.835	19.171
Escherichia-Shigella	0.222	0.066	0.161	0.429	0.009	2.122*	0.030	4.257	0.007	0.023
Clostridium	1.023	0.095*	0.882	0.097*	0.180	0.027*	0.388	0.050	0.265	0.009
Actinobacillus	1.008	0.788	0.263	1.089	0.001	0.240*	0.002	0.005	0.000	0.001
Blautia	0.642	0.336	0.953	0.654	2.820	0.252*	0.900	0.142	0.113	0.577
Rothia	0.913	1.536	0.956	0.872	0.527	1.407	0.104	0.421	0.002	0.002
Enterococcus	0.096	0.145	0.168	0.084	0.054	0.029	0.183	0.047	0.005	0.020
Parabacteroides	0.012	0.000	0.014	0.004	0.000	0.007	0.000	0.001	0.326	3.767
Prevotella	0.357	0.041*	0.184	0.047	0.004	0.007	0.002	0.001	1.874	0.698*
Mogibacterium	0.081	0.101	0.054	0.113	0.188	0.031	0.011	0.008	0.109	0.649
Pseudomonas	0.615	0.224	0.455	0.431	0.014	0.050	0.002	0.004	0.004	0.004
Corynebacterium	0.196	0.110	0.198	0.152	0.147	0.136	0.269	0.110	0.021	0.006
Acinetobacter	0.303	0.134	0.366	0.215	0.012	0.027	0.003	0.003	0.004	0.009
Bacillus	0.367	0.038*	0.756	0.034*	0.136	0.010*	0.036	0.005	0.006	0.009
Ruminococcus	0.045	0.037	0.140	0.107	0.036	0.006	0.025	0.004	0.221	0.059
Sharpea	0.054	0.005*	0.051	0.006*	0.040	0.001*	0.015	0.001	0.003	0.020
Haemophilus	0.210	0.011	0.121	0.013*	0.000	0.002*	0.000	0.000	0.000	0.000
Paracoccus	0.025	0.011	0.066	0.018	0.000	0.000	0.000	0.001	0.000	0.000
Stenotrophomonas	0.141	0.077	0.173	0.206	0.009	0.008	0.000	0.004	0.000	0.000
Rhizobium	0.117	0.047	0.097	0.082	0.008	0.023	0.001	0.002	0.000	0.001
Faecalibacterium	0.099	0.041	0.067	0.115	0.006	0.003	0.000	0.008	0.020	0.040
Solobacterium	0.051	0.040	0.027	0.034	0.028	0.012	0.019	0.001	0.113	0.019
Sphingomonas	0.035	0.013	0.048	0.019	0.066	0.053	0.002	0.032	0.003	0.002
Neisseria	0.044	0.000*	0.016	0.001	0.000	0.000	0.000	0.000	0.000	0.000
RC10_gut_group	0.004	0.000	0.008	0.000	0.000	0.002	0.000	0.000	0.086	0.044
Staphylococcus	0.053	0.030	0.068	0.071	0.064	0.023	0.004	0.005	0.001	0.001
Propionibacterium	0.037	0.033	0.035	0.048	0.001	0.000	0.000	0.001	0.000	0.000
Treponema	0.000	0.000	0.000	0.000	0.000	0.000	0.000	0.000	0.000	0.019*

Table S5. Genus-level effects of antibiotic treatment (Ant) on the dominant bacteria in the mucosa compared with the control group (Con). The data of relative abundance (%) are expressed as median. Asterisks indicate different from control group (Mann-Whitney *U*-test and a false discovery rate < 5%).

Items	Duodenum		Jejunum		Ileum	
	Con	Ant	Con	Ant	Con	Ant
Escherichia-Shigella	0.499	0.708	16.998	29.238	29.506	36.973
Alcaligenes	24.841	26.519	0.013	0.020	0.039	0.030
Prevotella	8.846	8.551	12.525	5.243	2.542	6.691
Actinobacillus	0.693	9.392	4.026	0.135*	6.849	15.622
Mycoplasma	0.041	0.028	4.082	21.619	7.019	0.239*
Phascolarctobacterium	2.432	1.860	0.502	0.565	0.429	0.741
Pseudomonas	0.307	0.622	7.454	2.144	3.136	1.010
Ruminococcus	3.846	3.007	1.642	2.217	0.905	2.224
Streptococcus	1.504	1.978	1.041	1.429	2.175	1.536
Blautia	2.807	3.447	3.500	1.498	0.732	2.177
Eubacterium	0.877	0.846	0.134	0.389	0.167	0.130
Faecalibacterium	1.628	1.879	1.195	1.410	0.192	0.724*
Lactobacillus	9.245	0.998	0.804	2.024	1.651	0.713
Clostridium	0.803	0.374*	0.886	2.525	19.158	2.894*
Coprococcus	2.008	1.796	0.892	1.274	0.535	0.966
Parabacteroides	0.761	0.719	0.413	0.123	0.085	0.414
Subdoligranulum	6.936	6.940	2.659	3.278	0.467	1.622
Achromobacter	1.615	1.099	0.023	0.002	0.018	0.002
Acidovorax	0.048	0.201	4.569	0.704*	1.326	0.411
Acinetobacter	0.056	0.153	1.371	0.912	0.469	0.357
Anaerovibrio	0.428	0.279	0.499	0.189	0.648	0.619
Bacteroides	0.782	0.624	0.314	0.185	0.152	0.474
Butyricicoccus	0.600	0.637	0.644	1.016	0.153	0.824
Campylobacter	2.232	2.370	1.386	0.575	0.187	1.194
Citrobacter	0.039	0.039	0.713	0.649	1.005	0.339
Leeia	4.335	2.935	0.069	0.078	0.109	0.127
Methylophilus	0.029	0.087	1.994	0.411*	0.673	0.287
Morganella	0.489	0.365	1.418	0.219	0.225	0.442
Ochrobactrum	6.247	5.883	0.009	0.000*	0.009	0.001
Terrisporobacter	0.310	0.149	0.107	0.441	7.066	1.036*

Table S6. Phylotype-level effects of antibiotic treatment (Ant) on the dominant bacteria in the lumen compared with the control group (Con). The data of relative abundance (%) are expressed as median. Asterisks indicate different from control group (Mann-Whitney *U*-test and a false discovery rate < 5%).

Items	Taxonomy	Stomach		Duodenum		Jejunum		Ileum		Colon	
		Con	Ant	Con	Ant	Con	Ant	Con	Ant	Con	Ant
OTU1762	g_*Streptococcus*	5.704	16.246	3.180	6.534	4.315	22.425*	6.574	6.569	0.002	0.011
OTU16	g_*Lactobacillus*	5.114	15.276	4.513	7.166	7.480	1.980	4.281	10.690	0.198	0.037
OTU1800	g_*Escherichia-Shigella*	0.396	0.119	0.293	0.417	0.015	4.008*	0.057	0.718	0.012	0.038
OTU1212	g_*Clostridium*	1.713	0.127*	1.593	0.096*	0.246	0.042*	0.682	0.303	0.190	0.011
OTU1757	g_*Lactobacillus*	3.401	0.260*	3.564	1.468	1.854	0.278	6.198	0.597	0.005	0.004
OTU218	g_*Subdoligranulum*	1.164	0.464	1.899	0.900	2.924	0.379*	0.575	0.184	11.479	2.163
OTU295	g_*Streptococcus*	0.545	0.659	0.631	0.795	2.340	0.893	3.671	0.853	0.051	0.138
OTU1174	g_*Streptococcus*	2.555	5.422	3.063	3.605	1.246	4.318	0.500	0.882	0.002	0.003
OTU478	g_*Subdoligranulum*	0.270	0.153	0.655	0.361	0.858	0.228*	0.319	0.046	3.083	0.809
OTU885	g_*Rothia*	1.628	2.683	1.704	1.608	0.893	2.298	0.187	0.790	0.003	0.003
OTU446	g_*Blautia*	1.029	0.585	1.445	1.139	4.336	0.428	1.219	0.236	0.140	0.656
OTU1392	g_*Lactobacillus*	0.884	0.409	1.066	0.781	1.354	0.085	0.480	0.584	0.022	0.009
OTU937	g_*Subdoligranulum*	0.079	0.314	0.135	0.665	0.152	0.179	0.040	0.094	0.080	0.658
OTU987	g_*Actinobacillus*	1.176	0.716	0.275	0.649	0.000	0.308*	0.000	0.000	0.000	0.000
OTU1751	f_*Peptostreptococcaceae*	0.997	0.223*	0.655	0.114*	0.344	0.048	0.218	0.065	0.477	0.007*
OTU1070	g_*Streptococcus*	0.470	2.669*	0.854	1.265	0.861	1.551*	0.211	1.179	0.000	0.000
OTU1865	f_*Erysipelotrichaceae*	0.582	0.054*	0.586	0.110*	0.614	0.025*	0.274	0.016	1.380	0.839
OTU554	g_*Actinobacillus*	0.591	0.610	0.122	1.238	0.000	0.247	0.003	0.009	0.000	0.000
OTU1618	g_*Enterococcus*	0.016	0.029	0.053	0.033	0.014	0.026	0.040	0.029	0.003	0.010
OTU188	p_*Proteobacteria*	2.546	1.174	1.679	0.207	0.346	0.076	0.015	0.010	0.000	0.000
OTU835	g_*Streptococcus*	0.436	0.062	0.834	0.033	3.968	0.117*	0.458	0.156	0.000	0.001
OTU73	f_*Lachnospiraceae*	0.027	0.019	0.051	0.078	0.134	0.018*	0.058	0.010	1.216	0.816
OTU979	p_*Tenericutes*	0.143	0.001*	0.073	0.013	0.043	0.000*	0.037	0.000*	0.067	0.009
OTU1890	g_*Mogibacterium*	0.115	0.185	0.077	0.147	0.199	0.052	0.021	0.012	0.197	1.069
OTU1643	g_*Parabacteroides*	0.001	0.000	0.008	0.005	0.000	0.002	0.000	0.001	0.139	1.713
OTU570	f_*Lachnospiraceae*	0.144	0.168	0.152	0.052	0.049	0.012*	0.186	0.000	0.033	0.180
OTU1607	g_*Corynebacterium*	0.208	0.146	0.105	0.080	0.217	0.084	0.398	0.191	0.014	0.000
OTU242	g_*Streptococcus*	0.515	0.214	0.609	0.197*	0.915	0.180	0.437	0.057	0.004	0.010
OTU592	f_*Lachnospiraceae*	0.533	0.283	0.458	0.284	0.662	0.072*	0.216	0.062	0.068	0.291
OTU1551	f_*Enterobacteriaceae*	0.744	0.288	0.595	0.629	0.015	0.011	0.003	0.010	0.003	0.004
OTU776	g_*Parabacteroides*	0.000	0.000	0.002	0.004	0.000	0.000	0.000	0.000	0.163	0.461
OTU24	g_*Pseudomonas*	0.574	0.249	0.409	0.456	0.018	0.003	0.000	0.003	0.003	0.004
OTU648	f_*Lachnospiraceae*	0.078	0.033	0.030	0.013	0.029	0.009	0.039	0.016	0.009	0.033
OTU1570	g_*Enterococcus*	0.147	0.232	0.242	0.104	0.077	0.025	0.264	0.032	0.003	0.010
OTU1325	g_*Acinetobacter*	0.423	0.224	0.464	0.335	0.015	0.007	0.000	0.004	0.001	0.006
OTU257	f_*Ruminococcaceae*	0.003	0.004	0.011	0.018	0.021	0.002	0.003	0.005	0.603	0.014*
OTU249	g_*Coprococcus*	0.532	0.400	0.699	0.103	0.522	0.036*	0.252	0.031	0.043	0.031
OTU192	p_*Tenericutes*	0.001	0.001	0.014	0.000	0.000	0.000	0.000	0.000	0.695	0.053*
OTU9	g_*Lactobacillus*	0.213	0.177	0.189	0.155	0.171	0.083	0.094	0.027	0.001	0.004
OTU810	g_*Veillonella*	0.114	0.039	0.074	0.059	0.006	0.008	0.003	0.003	0.000	0.000
OTU1188	g_*Collinsella*	0.095	0.033*	0.237	0.110	0.046	0.017	0.045	0.005	0.339	0.278
OTU423	p_*Tenericutes*	0.030	0.000*	0.012	0.000*	0.002	0.000	0.000	0.000	0.010	0.014
OTU1388	f_*Erysipelotrichaceae*	0.090	0.176	0.106	0.155	0.104	0.058	0.024	0.014	0.062	0.138
OTU767	g_*Atopobium*	0.153	0.069	0.242	0.079	0.125	0.018*	0.125	0.005*	0.316	0.135
OTU252	f_*Erysipelotrichaceae*	0.046	0.178	0.062	0.188	0.027	0.084	0.055	0.009	0.114	0.119
OTU384	g_*Bacillus*	0.584	0.069*	1.180	0.035	0.231	0.017	0.064	0.010	0.006	0.008

OTU499	f_*Ruminococcaceae*	0.043	0.003	0.024	0.007	0.017	0.004	0.006	0.000	0.005	0.014
OTU1088	g_*Lactobacillus*	0.170	0.021*	0.073	0.011*	0.225	0.024*	0.489	0.027	0.000	0.004
OTU734	g_*Collinsella*	0.005	0.000*	0.003	0.000	0.000	0.000	0.006	0.000	0.005	0.001
OTU989	g_*Sharpea*	0.100	0.009*	0.094	0.011*	0.068	0.000*	0.025	0.001	0.005	0.034

Table S7. Phylotype-level effects of antibiotic treatment (Ant) on the dominant bacteria in the mucosa compared with the control group (Con). The data of relative abundance (%) are expressed as median. Asterisks indicate different from control group (Mann-Whitney *U*-test and a false discovery rate < 5%).

Items	Taxonomy	Duodenum		Jejunum		Ileum	
		Con	Ant	Con	Ant	Con	Ant
OTU1800	g_*Escherichia-Shigella*	0.229	0.165	8.985	9.109	15.242	25.732
OTU491	g_*Alcaligenes*	25.602	23.065	0.010	0.008	0.000	0.023
OTU1680	g_*Mycoplasma*	0.005	0.010	0.502	2.144	0.735	0.006*
OTU987	g_*Actinobacillus*	0.202	0.392	0.349	0.035	0.368	0.879
OTU1212	g_*Clostridium*	0.490	0.271*	0.458	1.371	8.079	0.867*
OTU446	g_*Blautia*	2.106	2.834	0.996	0.665	0.219	0.749
OTU627	f_*Brucellaceae*	6.439	4.766	0.006	0.000*	0.004	0.000
OTU218	g_*Subdoligranulu*	4.510	1.283	0.720	0.877	0.197	0.182
OTU1085	g_*Pseudomonas*	0.179	0.272	4.027	0.431*	0.874	0.416
OTU554	g_*Actinobacillus*	0.132	0.188	0.388	0.003*	0.053	0.415
OTU1751	g_*Terrisporobacter*	0.203	0.096	0.059	0.295	2.409	0.304*
OTU959	g_*Prevotella*	1.266	1.105	1.048	0.366	0.092	0.300
OTU943	g_*Prevotella*	1.216	0.928	1.466	0.267	0.078	0.318
OTU570	g_*Ruminococcus*	1.267	0.351	0.327	0.508	0.180	0.108
OTU270	g_*Leeia*	2.188	1.764	0.017	0.069	0.004	0.049
OTU764	g_*Acidovorax*	0.039	0.076	3.050	0.214*	0.498	0.266
OTU249	g_*Coprococcus*	1.379	1.128	0.356	0.314	0.256	0.195
OTU1458	g_*Phascolarctobacterium*	1.292	1.298	0.290	0.442	0.025	0.251
OTU478	g_*Subdoligranulum*	1.848	1.612	0.257	0.282	0.019	0.117
OTU263	g_*Prevotella*	0.630	0.316	1.145	0.200	0.108	0.420
OTU16	g_*Lactobacillus*	0.514	0.468	0.083	0.307	0.268	0.243
OTU256	g_*Faecalibacterium*	1.012	1.663	0.625	0.721	0.087	0.537*
OTU2016	g_*Prevotella*	1.355	0.795	0.429	0.236	0.197	0.405
OTU1449	g_*Campylobacter*	0.068	0.118	0.081	0.113	0.015	0.144
OTU1392	g_*Lactobacillus*	0.126	0.059	0.110	0.182	0.149	0.093
OTU295	g_*Streptococcus*	0.396	0.605	0.484	0.415	0.623	0.264
OTU937	g_*Subdoligranulum*	0.499	0.484	0.312	0.037	0.050	0.211*
OTU1757	g_*Lactobacillus*	0.589	0.082	0.069	0.576	0.445	0.151
OTU1258	g_*Methylophilus*	0.021	0.044	1.223	0.219	0.336	0.172*
OTU1762	g_*Streptococcus*	0.405	0.741	0.166	0.014	0.146	0.411
OTU1920	g_*Prevotella*	0.057	0.375	0.000	0.020	0.000	0.000
OTU2005	g_*Butyricicoccus*	0.172	0.162	0.413	0.214	0.076	0.351
OTU1756	g_*Campylobacter*	0.048	0.030	0.025	0.000	0.001	0.038
OTU1228	g_*Prevotella*	0.458	0.221	0.446	0.048	0.013	0.150
OTU1150	g_*Achromobacter*	1.536	1.035	0.021	0.002	0.017	0.002
OTU1551	g_*Citrobacter*	0.030	0.020	0.711	0.228	0.220	0.169
OTU784	g_*Prevotella*	0.115	0.113	0.204	0.059	0.005	0.019
OTU24	g_*Pseudomonas*	0.028	0.028	0.388	0.162	0.427	0.200
OTU592	g_*Ruminococcus*	0.447	0.412	0.193	0.047	0.091	0.076
OTU1865	g_*Eubacterium*	0.634	0.234	0.078	0.087	0.073	0.052
OTU1396	g_*Brevundimonas*	0.051	0.040	0.162	0.211	0.128	0.145
OTU1323	f_*Ruminococcaceae*	0.461	0.159	0.149	0.155	0.109	0.147
OTU1325	g_*Acinetobacter*	0.023	0.028	0.631	0.210	0.209	0.169
OTU71	g_*Klebsiella*	0.019	0.010	0.196	0.191	0.161	0.126
OTU29	f_*Ruminococcaceae*	0.321	0.284	0.065	0.058	0.035	0.084
OTU526	g_*Bacteroides*	0.313	0.235	0.091	0.091	0.021	0.078

OTU746	g_*Barnesiella*	0.044	0.054	0.404	0.019	0.000	0.282*
OTU981	g_*Brucella*	0.012	0.018	0.298	0.168	0.108	0.164
OTU1240	f_*Ruminococcaceae*	0.070	0.197	0.311	0.064	0.062	0.206
OTU648	g_*Ruminococcus*	0.289	0.064	0.015	0.104	0.037	0.007

Table S8. Distribution of the KEGG categories predicted to be represented in bacterial communities of lumen of duodenum, jejunum and ileum compared with the mucosa. The data from control group were used for analysis here. Data are presented as mean relative abundances. Asterisks indicate different in lumen compared with the mucosa in the corresponding location (false discovery rate < 5%).

Items	KEGG Pathways	Duodenum		Jejunum		Ileum	
		Mucosa	Lumen	Mucosa	Lumen	Mucosa	Lumen
Cell Motility	Bacterial motility proteins	1.292	0.895*	1.240	0.885	1.325	0.833
Cell Motility	Flagellar assembly	0.630	0.171*	0.542	0.068*	0.612	0.289
Membrane Transport	Bacterial secretion system	0.822	0.562*	0.738	0.477*	0.702	0.490*
Folding, Sorting and Degradation	Ubiquitin system	0.003	0.021*	0.011	0.022	0.009	0.011
Amino Acid Metabolism	Alanine, aspartate and glutamate metabolism	1.000	0.893*	0.885	0.923	0.768	0.947*
Amino Acid Metabolism	Arginine and proline metabolism	1.253	1.155*	1.117	1.280*	1.002	1.047
Amino Acid Metabolism	Glycine, serine and threonine metabolism	0.822	0.708*	0.810	0.688*	0.729	0.715
Amino Acid Metabolism	Lysine biosynthesis	0.784	0.665*	0.682	0.679	0.563	0.732*
Amino Acid Metabolism	Lysine degradation	0.248	0.183	0.301	0.123*	0.245	0.116*
Amino Acid Metabolism	Phenylalanine metabolism	0.218	0.186	0.267	0.173*	0.214	0.135
Amino Acid Metabolism	Phenylalanine, tyrosine and tryptophan biosynthesis	0.793	0.665*	0.718	0.767	0.567	0.642
Amino Acid Metabolism	Tryptophan metabolism	0.286	0.231	0.341	0.170	0.279	0.151*
Amino Acid Metabolism	Valine, leucine and isoleucine biosynthesis	0.791	0.698*	0.701	0.777	0.686	0.708
Amino Acid Metabolism	Valine, leucine and isoleucine degradation	0.420	0.325	0.477	0.220*	0.382	0.220*
Carbohydrate Metabolism	Butyrate metabolism	0.681	0.599	0.783	0.468*	0.713	0.594
Carbohydrate Metabolism	Citrate cycle	0.605	0.466*	0.617	0.379*	0.523	0.372*
Carbohydrate Metabolism	Propionate metabolism	0.665	0.543*	0.700	0.425*	0.615	0.521
Carbohydrate Metabolism	Starch and sucrose metabolism	0.742	0.898*	0.785	0.988*	0.842	1.061*
Enzyme Families	Peptidases	1.675	1.925*	1.668	2.042*	1.641	1.842*
Glycan Biosynthesis and Metabolism	Glycosyltransferases	0.322	0.487*	0.346	0.513*	0.287	0.392
Glycan Biosynthesis and Metabolism	Lipopolysaccharide biosynthesis	0.253	0.174	0.298	0.152*	0.208	0.075

Glycan Biosynthesis and Metabolism	N-Glycan biosynthesis	0.020	0.050*	0.016	0.069*	0.008	0.024
Metabolism of Other Amino Acids	beta-Alanine metabolism	0.298	0.233	0.345	0.212*	0.248	0.163
Metabolism of Other Amino Acids	Taurine and hypotaurine metabolism	0.095	0.115*	0.114	0.098*	0.128	0.129
Metabolism	Carbohydrate metabolism	0.146	0.214*	0.169	0.236*	0.167	0.185
Metabolism	Energy metabolism	0.732	0.603*	0.857	0.598*	0.752	0.614

Table S9. Effect of antibiotic treatment (Ant) on the KEGG orthology involved in carbohydrate-active enzyme predicted to be differentially represented (false discovery rate < 5%) in bacterial communities compared with control group (Con). Data are presented as mean relative abundances. L represents for Lumen and M for mucosa.

Gut location	KEGG_Description	KEGG orthology	Cazy family	Con	Ant
Stomach-L	sialidase-1 [EC:3.2.1.18]	K01186	GH1	0.012	0.041
	beta-glucosidase [EC:3.2.1.21]	K05350	GH1	0.044	0.067
	hyaluronate lyase [EC:4.2.2.1]	K01727	PL8	0.005	0.021
	sucrose phosphorylase [EC:2.4.1.7]	K00690	GH13	0.016	0.033
	endo-1,3(4)-beta-glucanase [EC:3.2.1.6]	K01180	GH9	6.45E-06	0
Duodenum-L	pectate disaccharide-lyase [EC:4.2.2.9]	K01731	PL1	4.61E-06	0
Jejunum-L	xylan 1,4-beta-xylosidase [EC:3.2.1.37]	K01198	GH1	0.000	0.002
	(1->4)-alpha-D-glucan 1-alpha-D-glucosylmutase [EC:5.4.99.15]	K06044	GH13	0.000	0.002
	maltooligosyltrehalose trehalohydrolase [EC:3.2.1.141]	K01236	GH13	0.000	0.002
	alpha-N-acetylglucosaminidase [EC:3.2.1.50]	K01205	GH89	0.000	0.001
	trehalose-phosphatase [EC:3.1.3.12]	K01087	GT20	0.000	0.002
	alpha,alpha-trehalose-phosphate synthase (UDP-forming) [EC:2.4.1.15]	K00697	GT20	0.000	0.003
Ileum-M	4-alpha-glucanotransferase [EC:2.4.1.25]	K00705	GH13	0.048	0.065
	levanase [EC:3.2.1.65]	K01212	GH32	0.003	0.011
	alpha-L-fucosidase [EC:3.2.1.51]	K01206	GH29	0.014	0.034
	starch synthase [EC:2.4.1.21]	K00703	GT5	0.048	0.065
Ileum-L	levansucrase [EC:2.4.1.10]	K00692	GH32	4.73E-05	0.000
Colon-L	beta-glucosidase [EC:3.2.1.21]	K01188	GH1	0.069	0.045
	endo-1,4-beta-xylanase [EC:3.2.1.8]	K01181	GH3	0.019	0.005
	endoglucanase [EC:3.2.1.4]	K01179	GH5	0.079	0.027
	cellulose 1,4-beta-cellobiosidase [EC:3.2.1.91]	K01225	GH5	0.000379	8.51E-05
	molybdenum transport protein [EC:2.4.2.-]	K03813	GH10	0.006	0.002
	alpha-N-arabinofuranosidase [EC:3.2.1.55]	K01209	GH13	0.049	0.024
	levansucrase [EC:2.4.1.10]	K00692	GH32	0.005	0.000128
